# The Effect of Selective Laser Melting Fabrication Parameters on the Tensile Strength of an Aged New Maraging Steel Alloy with 8% Cr, Reduced Ni Content (7%), and No Co or Mo

**DOI:** 10.3390/ma16217008

**Published:** 2023-11-01

**Authors:** Inés Pérez-Gonzalo, Alejandro González-Pociño, Florentino Alvarez-Antolin, Laura del Rio-Fernández

**Affiliations:** 1 Materials Science and Metallurgic Engineering Department, University of Oviedo, Independencia 13, 33004 Oviedo, Spain; uo225790@uniovi.es (I.P.-G.); alvarezflorentino@uniovi.es (F.A.-A.); 2 ArcelorMittal Global R&D Asturias, P.O. Box 90, 33400 Avilés, Spain; laura.delriofernandez@arcelormittal.com

**Keywords:** maraging steel, selective laser melting, balling, laser power, scanning speed, porosity

## Abstract

The aim of this paper was to optimise the manufacturing parameters of a new maraging steel alloy with 8% Cr, reduced Ni content (7%), and no Co or Mo. This alloy was developed by ArcelorMittal and its trade name is LeanSi. The alloy was produced using the selective laser melting (SLM) process. In the as-built state, the microstructure of the alloy was fully martensitic. The optimisation of the manufacturing parameters was determined via a multivariate factorial design of experiments including 12 experiments and three factors. The factors (i.e., the fabrication parameters) analysed were laser power, scanning speed, and hatch distance. The objective was to eliminate porosity and maximise density. It was concluded that, to achieve this, the laser power should be set at 250 W, the scanning speed at 1000 mm/s, and the hatch distance at 80 microns. The porosity obtained under these manufacturing parameters was 0.06 ± 0.03% with a confidence level of 95%. If these manufacturing parameters were modified, the material exhibited a defective interlayer bond with the formation of “balling” and high porosity. The tensile specimens tested in the as-built state showed plastic deformation. However, all the aged specimens showed brittle fracture behaviour, evidenced by the presence of very small micro-cavities (where the fracture energy consumed was very small) and small cleavage planes. The specimens produced with the manufacturing parameters at their optimum levels and aged at 480 °C for 2 h achieved tensile strength values that averaged 1430 MPa. The porosity of these specimens was reduced by more than 85%. Reverse austenite was detected at ageing temperatures of 540 °C upwards.

## 1. Introduction

Maraging steels show excellent mechanical strength because of their microstructure. These steels have a martensitic matrix with nanoprecipitates formed after an ageing treatment. The alloying elements used in these steels are mainly Ti, Al, Mo, and Co [1,2,3,4]. These steels are widely used in applications that require high strength and durability, such as the aeronautical industry, tool manufacturing, or in components used in nuclear reactors [5,6,7]. Structurally hardening precipitates have a coherent interface with the matrix and increase the mechanical strength of these steels due to their interaction with dislocations [2]. The main precipitates that promote structural hardening of these steels are Ni_3_(Ti, Mo) or Fe_2_Mo-Laves types [8,9,10,11,12]. Titanium is the most effective strengthening agent in these steels. When manufacturing these steels, the carbon content should be reduced as much as possible to prevent the formation of titanium carbides [13]. The Laves phases have AB_2_ stoichiometry of the (Ti, Mo)(Fe, Cr, Ni)_2_ type. These precipitates remain dispersedly integrated in the martensite laths after cooling [2,13,14]. These precipitates have a compact hexagonal crystalline symmetry (HCP) and their size is around 200 nm [6,15]. They form at around 550 °C and do not dissolve completely until above 900 °C [14]. Laves precipitates consume Ti and Mo atoms, so that if the austenitisation process occurs at lower temperatures (e.g., 800–900 °C) the precipitation hardening potential can be reduced [14]. The austenite formed during the ageing process is known as inverse or reverse austenite. The amount of this austenite formed during ageing increases with higher ageing temperatures and longer ageing times [2,16]. The formation of reverse austenite is due to the matrix being enriched with elements that stabilise austenite [17], such as those resulting from the partial dissolution of Ni_3_(Ti, Mo) and the formation of Fe_2_Mo [18]. The surrounding matrix is enriched in Ni, which leads to partial reversion of the austenite [19]. Reverse austenite is remarkably stable and does not transform during cooling after ageing [20,21,22,23]. Conventional martensitic steels with 18% Ni (maraging 300) harden mainly via the formation of Ni_3_Ti, in combination with Laves precipitates [10]. These steels are particularly costly to manufacture, mainly due to the expense of alloying elements such as Ni and Co [6]. Maraging 300 steels manufactured with traditional casting processes reach ultimate tensile stress values in the range of 1800–2000 MPa [24,25,26]. The values achieved in maraging 300 steels manufactured using SLM technology are in the range of 1400–2000 MPa [12,27,28]. SLM-manufactured maraging 300 alloys with a higher Ti content (1.17 wt.%) show better ageing potentials, reaching tensile strength values slightly above 2000 MPa (2057 MPa) and a ductility over 17% after ageing at 490 °C for 6 h. This higher Ti content favours the formation of reverse austenite [29]. Recent research [28,30] carried out on the influence of SLM fabrication parameters on the microstructure and mechanical properties of maraging 300 steels has found these parameters achieve mechanical properties comparable to those of the wrought 18Ni300.

The company ArcelorMittal (Arcelormittal Global R&D) has developed an alloy with 8% Cr, reduced Ni content (7%), and no Co or Mo, which considerably reduces its manufacturing cost. The trade name of this alloy is LeanSi. This alloy is manufactured with the selective laser melting (SLM) process and has Ni_3_Ti as the only structural hardening agent. The Ti content of LeanSi is 1%, which results in a high ageing hardening potential [29]. The SLM manufacturing process is part of the powder bed fusion (PBD) processes, which are additive manufacturing processes (AMs) [7,31,32,33,34,35]. Using SLM technology enables the manufacturing of tailor-made components [36,37,38]. The usage of this technology involves physical phenomena, such as melting, a complex heat transfer process, and the solidification of the melted metal powder [39]. The stability of the molten pool during the SLM process has significant effects on the microstructure and mechanical properties of the resulting material [40]. The laser beam heats the powder bed to melt and fuse the particles. This process involves different physical and thermal phenomena [40]. As a consequence of this heating, residual stresses appear in the material due to the large thermal gradients involved [41,42,43,44]. The factor most likely to cause defects in the interlayer bond is balling [45,46,47,48]. If balling occurs in a previously solidified layer, thus making the surface of this layer irregular and corrugated, it will lead to a significant variation in the thickness of the next deposited powder layer [40,49]. Other defects such as high porosity, unmelted powder, and gas entrapment can also occur in alloys manufactured using the SLM process. All the above-mentioned defects generate anisotropic material properties [7]. The combined effect of inadequate scanning parameters and poor powder melting can deteriorate the mechanical properties of the alloy [40]. To eliminate the bond defect and form a solid bond between two consecutive layers, a stable melt pool created under optimised fabrication parameters is essential, as this promotes sufficient partial remelting of the previous layer [40]. The main fabrication parameters of the process used to produce LeanSi are laser power (LP), layer thickness (LT), scanning speed (SS), and hatch distance (HD) [46]. These fabrication parameters allow the process to be characterised in terms of energy density [3,4,12,40,45,50]. Due to the planar motion of the heat source and the uniaxial motion of the build plate, achieving both a homogenised microstructure and isotropic mechanical properties in the printed martensitic steel is a challenge [7]. In this paper, we present the following information:

1. LeanSi steel is introduced. This new alloy has mechanical properties comparable to those of conventional M300 steels, but with a reduced cost. It was developed by ArcelorMittal and manufactured using the SLM process, with 8% Cr, a reduced Ni content (7%), and without Co or Mo.
2. Bond defects generated during the manufacturing process are analysed.
3. Experiments are carried out to optimise the fabrication parameters used in the manufacturing process of this alloy, with the aim of producing a defect-free material.

## 2. Materials and Methods

The material components used in this paper are shown in Table 1.

**Figure 1:**
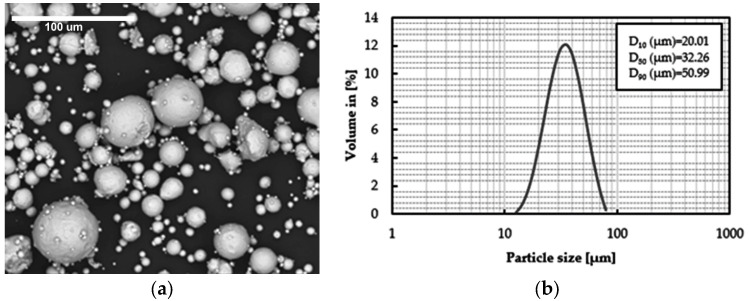
Powder used in the SLM process. (**a**) Scanning electron microscope (SEM) image of a random sample of the powder; (**b**) particle size distribution.

**Figure 2:**
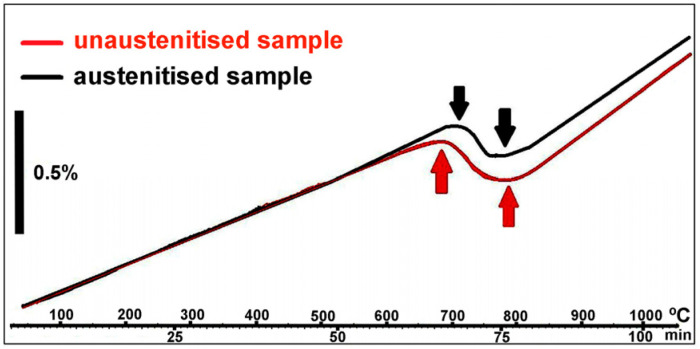
Dilatometric tests. From 800 °C onwards the material is fully austenitised. Heating rate 10 °C/min.

Several tensile specimens were melted in a RenAM500Q selective laser melter (RENISHAW, Wotton-under Edge, UK) using fabrication parameters which were later found to be inadequate. Figure 1 shows the geometry of the powder used. The average size of the powder was 32.26 µm (D_50_). The 10th percentile of powder size was 20.01 µm (D_10_) and the 90th percentile of powder size was 50.99 µm (D_90_). These specimens were aged with and without previous austenitisation and then tensile-tested. The austenitisation temperature was chosen based on a dilatometric test, using a Mettler-Toledo TGA/SDTA851 dilatometer (Mettler-Toledo, Barcelona, Spain). Figure 2 shows the results of this test. For both an as-printed sample and a sample previously austenitised at 1000 °C, the transformation from ferrite to austenite occurred in the 700–800 °C range. At 800 °C and above, the microstructure was fully austenitised. If the material undergoes austenitisation at temperatures below 900 °C, the Laves precipitates present in the material negatively affect its ageing potential. For this reason, the austenitised specimens were treated at 1000 °C for 20 min and then water-quenched [14]. Since the optimum ageing temperature was unknown, a wide range of ageing temperatures in the range of 460–580 °C was tested. The ageing time was 2 h in all cases.

A multilevel factorial design of experiments including 3 factors and 12 experiments using STATGRAPHICS Centurion XVI (The Plains, VA, USA) was developed to optimise the fabrication parameters and therefore minimise porosity and maximise material density [51,52,53,54].

These experiments were designed to deliberately modify the conditions normally used during fabrication and investigate how this resulted in changes to the responses under study (e.g., the porosity-volume fraction). The factors (i.e., fabrication parameters) that were modified were previously selected [55,56,57]. An effect of these modifications is defined as significant if it is unlikely to be the result of chance. To determine this, a significance test is performed for each effect using the hypothesis test with t-Student distribution. A bilateral hypothesis test is used with a significance level of 5% (α = 0.05), where the null hypothesis is to consider that the mean of each effect is 0 [54,57,58]. If an analysis provides sufficient evidence to reject this hypothesis, it can be considered significant at a confidence level of 95%. Table 2 shows the factors and levels analysed and Table 3 shows the matrix of experiments. The design was carried out using 2 replications of each experiment [59]. In all cases, the LT was 50 μm. The laser scanning sequence used was as follows: orthogonal scanning, cross scanning, and S-scanning. The printer used includes a 200 W fibre laser (Yb: YAG, wavelength: 1075 nm, SPI).

Optical microscopy and scanning electron microscopy were used to analyse the fracture facies after tensile tests. For this purpose, a Leica TCS-SP8X spectral confocal laser microscope (Leica Microsystems, Wetzlar, Germany), a Leica M205FA fluorescence stereomicroscope (Leica Microsystems, Wetzlar, Germany), and a JEOL JSM-5600 scanning electron microscope (SEM), equipped with the characteristic X-ray scattering microanalysis system (JEOL, Nieuw-Vennep, The Netherlands), were used. The weight percentage of austenite was determined by means of X-ray diffraction with a Cu X-ray tube. The diffractometer used was a PANalytical X’Pert Pro MPD (PANalytical B.V., Almelo, The Netherlands). All this equipment belongs to the Scientific and Technical Services of the University of Oviedo. The tensile test was performed according to the UNE-EN-ISO 6892 standard [60], using an Instron 5582 (Instron, Norwood, MA, USA) with a displacement rate of 5 mm/min and a load limit of 100 kN [61,62]. Porosity quantification was performed via optical microscopy using Image J v1.46r image processing software (NIH Image J Software, Bethesda, MD, USA) by analysing 20 micrographs for each experiment and performing 2 replicates [63]. The optical microscope used was a Nikon Epiphot 200 (Nikon, Tokyo, Japan). Densities were calculated according to Archimedes’ principle [27,41,43].

## 3. Results and Discussion

Table 4 shows the weight percentages and lattice parameters of the single crystalline phase, detected using X-ray diffraction, in the specimens with and without prior austenitisation. These results confirmed that the new LeanSi alloy had no retained austenite in the as-printed state; thus, it can be aged without prior austenitisation treatment (AT).

**Figure 3:**
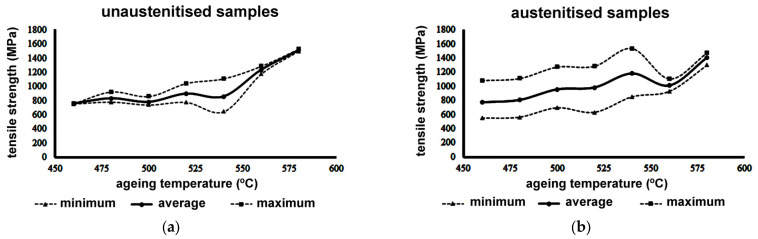
Ultimate tensile strength of samples aged at different temperatures: (**a**) heat-treated samples at different ageing temperatures without AT; (**b**) heat-treated samples at different ageing temperatures with AT at 1000 °C followed by water quenching. The maximum tensile stress in the as-printed sample was 932 MPa.

Figure 3 shows the ultimate tensile stresses of the specimens aged at different temperatures. The results exhibited a wide variability and uncertainty, which made it impossible to establish a reliable value. This was later found to be due to randomly distributed bonding defects, which occurred between layers during the manufacturing process of the samples.

**Figure 4:**
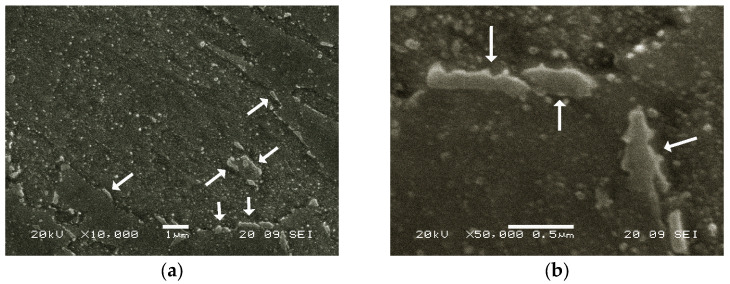
Sample overaged for 48 h at 580 °C. (**a**) ×10,000, (**b**) ×50,000. Arrows mark the presence of reverse austenite.

All tensile fractures observed on the aged samples occurred in the elastic range, except for those in specimens aged at 580 °C, which showed a slight plastic deformation before the fracture. This suggests that, at this temperature, reverse austenite began to form. Figure 4 shows a sample overaged for 48 h at 580 °C. A significant presence of reverse austenite was observed in this sample along with a high density of structural hardening precipitates, identified as Ni_3_Ti in the equilibrium state.

**Figure 5:**
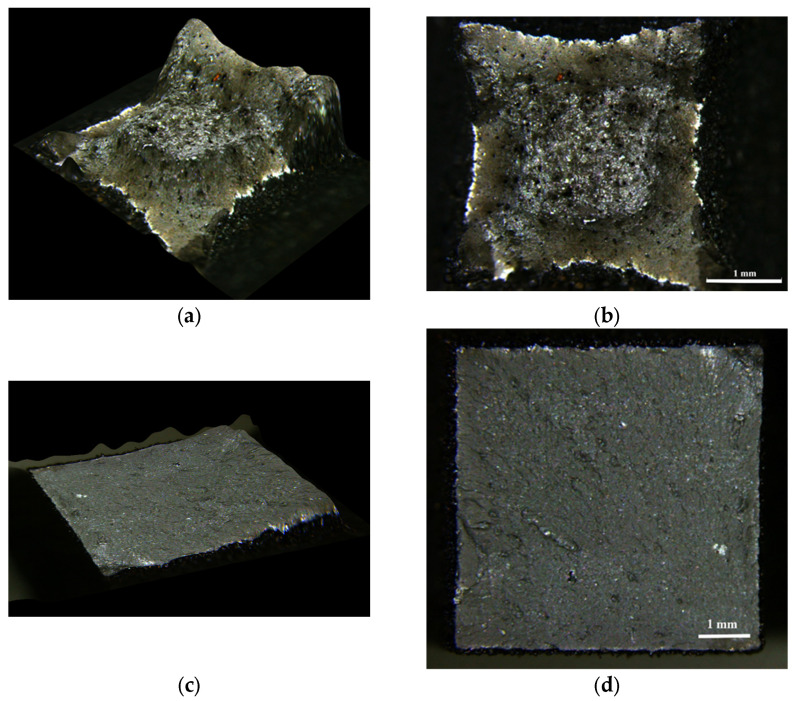
Representative fracture facies of broken tensile specimens: (**a**,**b**) austenitised, unaged specimen; (**c**,**d**) non-austenitised specimen aged at 540 °C.

Due to the high variability of the tensile test results, the fracture facies were analysed. Figure 5 shows the fracture facies of two of the broken tensile specimens after the AT at 1000 °C.

Figure 5a,b show the facies without the subsequent ageing treatment. Figure 5c,d show the fracture facies of samples aged at 540 °C. The unaged sample exhibited plastic deformation, linked to a ductile fracture, where distinctive dimples can be seen [37,61]. In comparison, the aged specimen broke without plastic deformation, showing brittle fracture facies [37]. All the specimens aged up to 540 °C showed this behaviour. However, a slight plastic deformation was observed in samples previously aged at 580 °C, which was attributed to the fact that the process of precipitation of reverse austenite started at this temperature.

**Figure 6:**
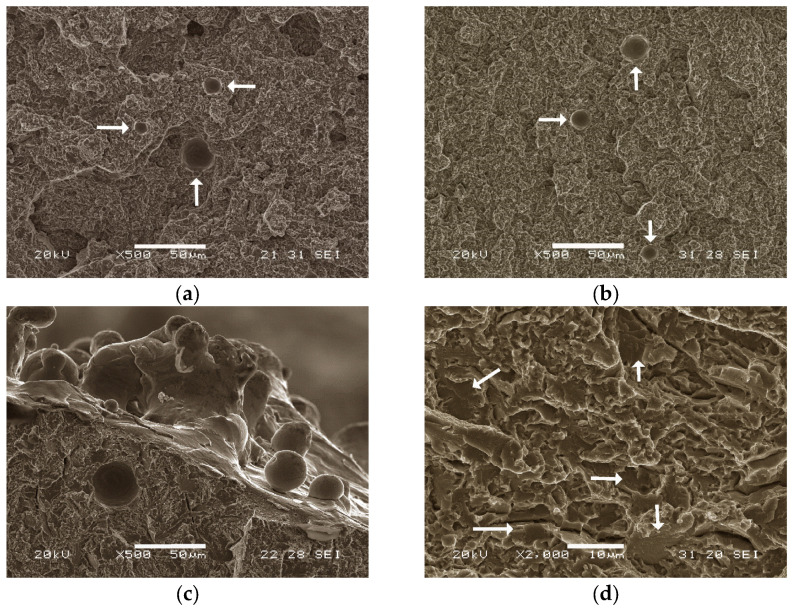
Fracture facies of specimens aged at 540 °C: (**a**,**c**) samples without AT; (**b**,**d**) samples with AT. (**a**,**b**) arrows indicate the voids left by atomised particles that did not melt; (**d**) arrows point to the small cleavage planes characteristic of brittle fracture behaviour.

Figure 6 shows the fracture facies of two specimens aged at 540 °C, one of them (Figure 6a,c) without the AT and the other one (Figure 6b,d) with the AT. Voids left by atomised particles that did not melt during the SLM process can be observed. Despite showing no signs of plastic deformation, the fracture facies showed “fibrous” regions with very small microcavities, where the fracture energy consumed was very small. Figure 6d highlights the multiple cleavage planes with very small surface areas, typical of a brittle mechanism fracture.

**Figure 7:**
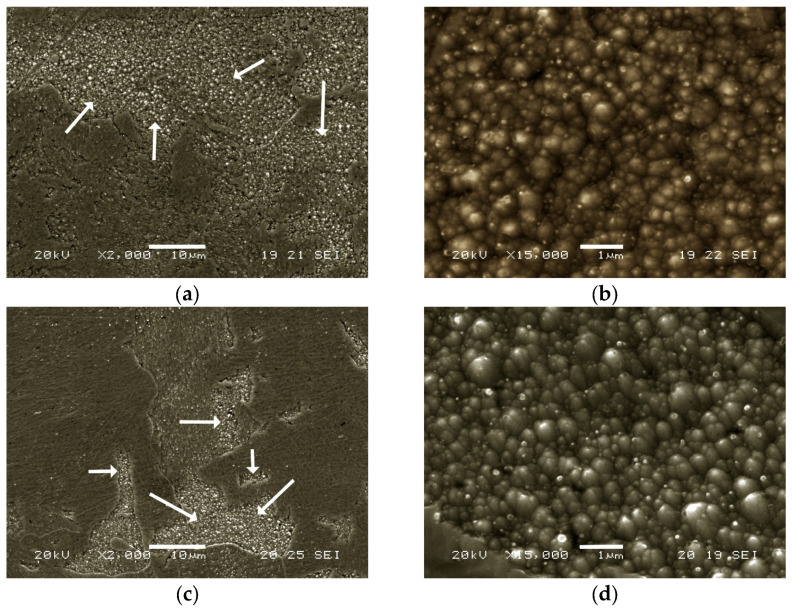
(**a**,**b**) Samples in the as-printed state; (**c**,**d**) austenitised samples. Arrows in (**a**,**c**) indicate the balling areas; (**b**,**d**) close up view of the balling areas.

Figure 7 shows the microstructure in the as-printed state and after the AT at 1000 °C. In both cases, large areas of balling are visible. Balling is a defect that occurs when the molten material does not wet the underlying substrate due to surface tension, and instead tends to spheroidise the liquid. It is essential to determine appropriate fabrication parameters to avoid this defect [47].

If the sample has internal defects (e.g., porosity, weak interlayer bonds, and/or balling), these will have a random location and a variable size distribution. Consequently, erroneous results in terms of the mechanical properties (e.g., tensile strength) will be obtained, as shown in Figure 3. It should be taken into consideration that tensile fractures are caused by unstable propagation of the larger, or weaker, bonding defect. In addition, the existing pores form zones of high stress concentration, which promote crack initiation and propagation [40]. The proposed design of experiments (Table 2 and Table 3) aimed to determine which of the fabrication parameters significantly influence the observed defects and, consequently, improve the quality of the manufactured SLM material. For this purpose, the effect of these fabrication parameters on the material’s porosity and density was analysed. Table 5 shows the results obtained in the porosity analysis and Table 6 reports the results obtained in the density analysis. The average porosity value across 12 experiments with two samples per experiment and three repetitions per sample was 0.472%.

**Figure 8:**
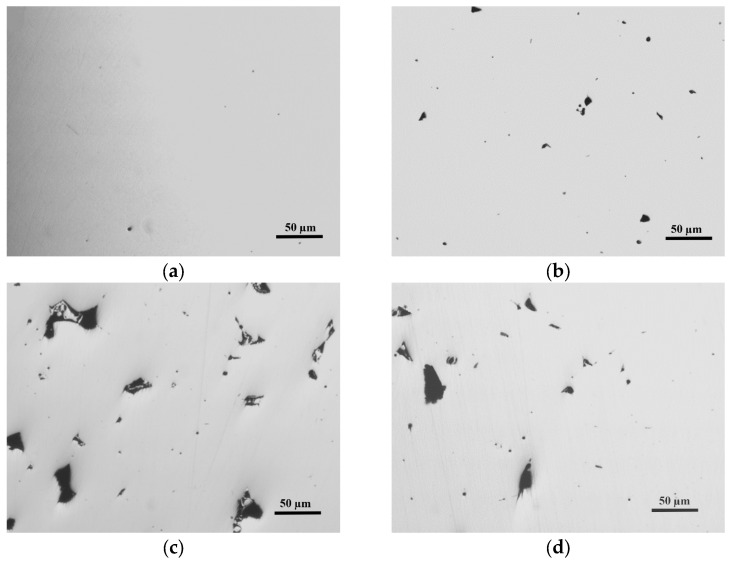
Micrographs of the polished-state samples: (**a**) Experiment 2; (**b**) Experiment 3; (**c**) Experiment 7; (**d**) Experiment 8.

**Figure 9:**
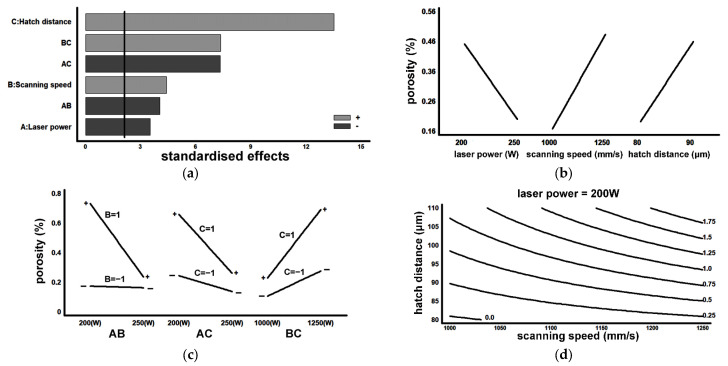
Graphical results for porosity: (**a**) Pareto plot of standardised effects; (**b**) main effects; (**c**) effects of 2nd-degree interactions; (**d**) contour lines with LP set at 200 W (porosity in %vol.).

**Figure 10:**
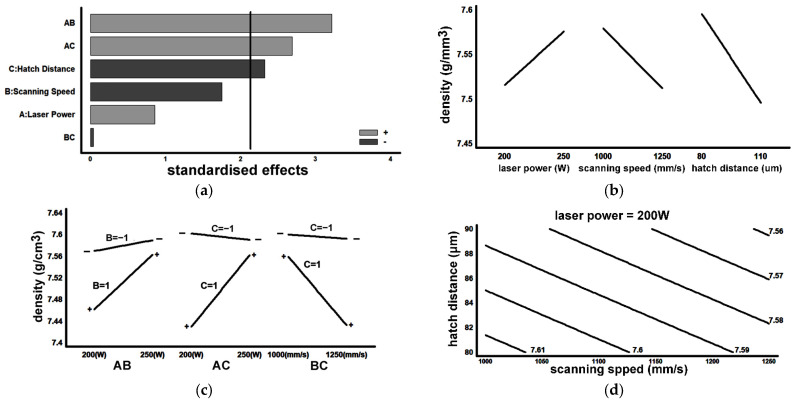
Graphical density results: (**a**) Pareto plot of standardised effects; (**b**) main effects; (**c**) effects of 2nd-degree interactions; (**d**) contour lines with LP set at 200 W (density values in g/cm^3^).

Figure 8 shows four representative polished-state samples that were analysed to determine the porosity of the material (%). Figure 9 and Figure 10 show the results obtained, respectively, in the porosity and density analyses. Figure 9a and Figure 10a show the Pareto plot on the standardised effects. The vertical lines mark the limit (in both cases this was 2.05) that each effect must exceed to be considered significant, tα/2. All the main factors and the second-order interactions were found to have a significant effect on porosity and density. Figure 9b,c and Figure 10b,c demonstrate that, to minimise the porosity and maximise the density, the SLM fabrication parameters that should be used are as follows: LP = 250 W, SS = 1000 mm/s, and HD = 80 μm. Figure 9d and Figure 10d, respectively, show an estimation of the contour lines of the porosity (%) and the density of the material for an LP set to 200 W. Both porosity and density showed their best results (i.e., lowest porosity and highest density) when SS was set at 1000 mm/s and HD was set at 80 μm.

Twenty-four samples with the SS set at 1000 mm/s and the HD set at 80 μm were fabricated to determine whether the use of these fabrication parameters improved the material. Of these 24 samples, 12 used LP set at 200 W and 12 used LP set at 250 W. Table 7 shows the porosity percentages obtained with a confidence level of 95%. Samples fabricated using LP = 250 W had porosity percentages below 10%. The average porosity of the 12 experiments with three replicates, shown in Table 5, was 0.472%. This can be compared with recent research on maraging 300 steels, manufactured using SLM technology, which showed porosities around 0.22% [3].

**Figure 11:**
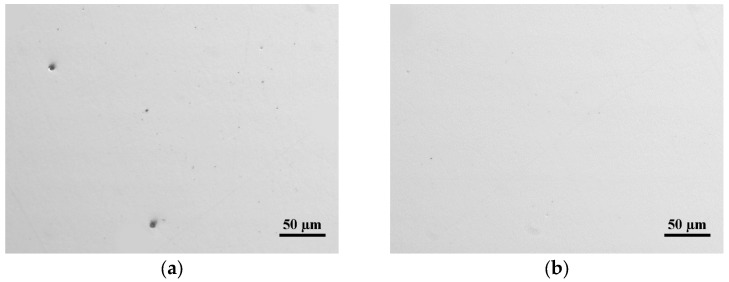
Micrographs in polished state; (**a**,**b**) porosity of specimens made using LP = 250 W.

Figure 11 shows two representative microstructures of localised porosity in the specimens fabricated using an LP = 250 W.

Table 8 shows a comparison between the true ultimate tensile strength of the samples aged at 480 °C for 2 h, fabricated according to the above-mentioned optimum fabrication parameters (SS = 1000 mm/s and HD = 80 microns), for LPs of 200 and 250 W. In all cases, fractures after tensile tests occurred without plastic deformation (in the elastic range). These results can be compared with the homologous specimens (aged at 480 °C) manufactured using the manufacturing parameters set at their previous (non-optimal) levels. The values achieved with the homologous specimens can be seen in Figure 3. The average values for tensile strength obtained for non-austenitised specimens were 832 MPa and 805 MPa for austenitised specimens. In austenitised samples, the interval between the maximum and minimum values was very wide due to the randomness of the manufacturing defects in the specimens. The improvement in results shown in Table 8 is evident, reaching a maximum tensile strength of 1430 MPa with an LP = 250 W. Although these values are slightly lower than those achieved with maraging 300 steels produced with traditional casting and SLM processes, they encourage further research to find a heat treatment that will achieve even higher tensile strength values.

**Figure 12:**
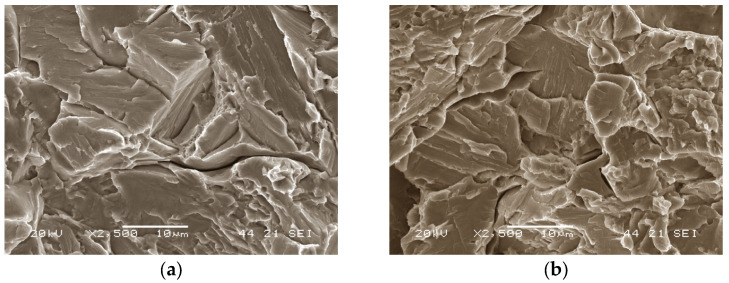
Fracture facies of two specimens fabricated using 250 W laser power and aged at 480 °C for 2 h, (**a**,**b**) showing the same fracture behaviour.

Figure 12 shows the representative fracture facies of specimens manufactured with the fabrication parameters at their optimum levels and aged at 480 °C for 2 h. Brittle cleavage fracture facies were observed. This means that any microstructural defect (e.g., porosity, weak joints between layers, and/or balling) will progress rapidly under tensile stresses without the material being able to offer an energetic obstacle, through plastic deformation, to prevent this. It is therefore essential to prevent this type of defect from occurring during the processing of the material.

## 4. Conclusions

The aim of this study was to optimise the manufacturing parameters of the selective laser melting (SLM) process for a new maraging steel alloy with 8% Cr, reduced Ni content, and no Co or Mo. The optimisation was conducted by applying a multivariate factorial design of experiments, including 12 experiments and three factors, with the aim of minimising porosity and maximising density. From these experiments it was concluded that, to achieve this aim, the LP should be set at 250 W, the SS at 1000 mm/s, and the HD at 80 μm. Tensile specimens tested in the as-printed state showed plastic deformation, whilst all aged specimens showed brittle fractures. The specimens produced by setting the fabrication parameters to their optimum levels, and aged at 480 °C for 2 h, reached average true ultimate tensile strength values of 1430 MPa. The porosity was reduced by more than 85% compared with previous manufacturing parameters, reaching values of 0.06 ± 0.03%, with a confidence level of 95%. Reverse austenite was detected for ageing temperatures of 540 °C upwards.

## Figures and Tables

**Table 1:** Chemical composition of the powder used in the SLM process (wt.%).

Cr	Ni	Si	Ti	Cu	Fe
8	7	1	1	1	Bal.

**Table 2:** Factors and levels.

Factors			Levels		
Code	Description of the Factors	Units	Level −1	Level 0	Level 1
A	Laser power (LP)	W	200	--	250
B	Scanning speed (SS)	mm/s	1000	--	1250
C	Hatch distance (HD)	μm	80	90	110

**Table 3:** Matrix of experiments.

Experiment	A	B	C	Restricted Confusion Pattern
1	−1	−1	−1	Factor A Factor B Factor C Interaction AB Interaction AC Interaction BC
2	1	−1	−1	
3	−1	1	−1	
4	1	1	−1	
5	−1	−1	1	
6	1	−1	1	
7	−1	1	1	
8	1	1	1	

**Table 4:** XRD analysis results.

Sample	Rietveld Adjustment	Phase	a (Å)	wt. %
Without austenitisation treatment (without AT)	Rwp = 11.8 Chi2 = 1.82	Ferrite	2.874	100
With austenitisation treatment (whit AT)	Rwp = 11.8 Chi2 = 2.36	Ferrite	2.874	100

**Table 5:** Porosity results (%).

Experiment	Replicate 0	Replicate 1	Replicate 2	Effects	
1	0.12	0.19	0.14		
2	0.06	0.07	0.06		
3	0.34	0.36	0.32		
4	0.21	0.23	0.2	−0.45	A
5	0.19	0.16	0.17	0.52	B
6	0.08	0.09	0.09	0.85	C
7	0.42	0.41	0.37	−0.17	AB
8	0.26	0.25	0.24	−0.46	AC
9	0.75	0.71	0.74	0.46	BC
10	0.22	0.19	0.24		
11	2.24	2.26	2.23		
12	0.80	0.7	0.9		

**Table 6:** Density results (g/cm^3^).

Experiment	Replicate 0	Replicate 1	Replicate 2	Effects	
1	7.63	7.61	7.60		
2	7.57	7.61	7.58		
3	7.59	7.59	7.57		
4	7.61	7.57	7.60	0.055	A
5	7.58	7.61	7.57	0.057	B
6	7.59	7.59	7.59	0.097	C
7	7.56	7.56	7.56	0.03	AB
8	7.61	7.59	7.59	0.072	AC
9	7.53	7.51	7.53	−0.062	BC
10	7.60	7.60	7.60		
11	7.35	7.32	7.35		
12	7.53	7.55	7.50		

**Table 7:** Percentage of porosity at 95% confidence level.

Laser Power (LP)	State	Porosity (%)
200 W	As-printed	0.16 ± 0.04
250 W	As-printed	0.06 ± 0.03

**Table 8:** True ultimate tensile strength (MPa).

Laser Power (LP)	State	Ageing	N° Trials	Average (MPa)
200 W	As-printed	480 °C; 2 h	3	1201
250 W	As-printed	480 °C; 2 h	3	1430

## Data Availability

Data are contained within the article.
